# Methodological adjustments for experimental studies including neurodiverse participants: A checklist for before, during, and after laboratory visits

**DOI:** 10.1016/j.mex.2024.102658

**Published:** 2024-03-10

**Authors:** Anne-Laure Le Cunff, Caitlin Glover, Brandon-Lee Martis, Vincent Giampietro, Eleanor Dommett

**Affiliations:** a Institute of Psychiatry, Psychology & Neuroscience, King's College London, 16 De Crespigny Park, London SE5 8AB, United Kingdom; b School of Engineering and Informatics, University of Sussex, Brighton, BN1 9RH, United Kingdom

**Keywords:** Participatory research, Inclusive research, Neurodiversity, Neuroscience, Checklist for Experimental Studies Including Neurodiverse Participants

## Abstract

In this paper, we developed an experimental checklist for laboratory experiments including neurodiverse participants, particularly those with attention deficit hyperactivity disorder (ADHD), autism spectrum disorder (ASD), and dyslexia. The checklist suggests additions to the basic requirements for ethical laboratory-based studies with human participants. The suggestions emphasize physical comfort, the agency of participants concerning environmental adjustments, clarity of communication, and a focus on participants’ overall well-being. Those methodological guidelines aim to help researchers in facilitating inclusive and accessible laboratory environments for neurodiverse participants in order to:

• Enhance research validity by minimizing the influence of factors that affect responses in neuroscience experiments.
• Facilitate research recruitment by encouraging continued participation in future studies and increasing word-of-mouth.
• Improve research dissemination by fostering a more positive perception of the research process amongst neurodiverse individuals and encouraging community involvement.

## Graphical abstract

**Figure:**
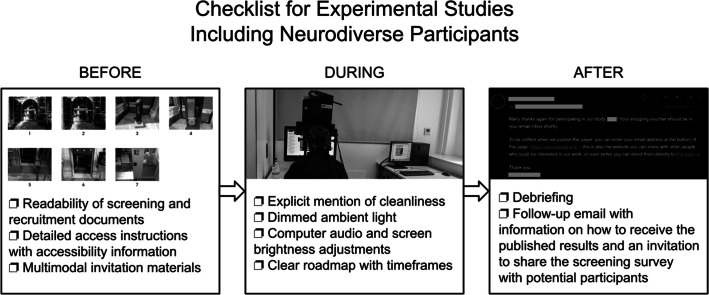

Specifications table

**Table:** 

Subject area:	Neuroscience
More specific subject area:	Cognitive Neuroscience
Name of your method:	Checklist for Experimental Studies Including Neurodiverse Participants
Name and reference of original method:	Rios, D., Magasi, S., Novak, C., & Harniss, M. (2016). Conducting accessible research: including people with disabilities in public health, epidemiological, and outcomes studies. *American journal of public health, 106*(12), 2137-2144.
Resource availability:	The checklist is available for download as a PDF file in the supplementary material section of this paper.

## Method details

Neurodiversity is a framework for understanding the innate variety in human cognition and brain function [4]. Initially adopted by the autistic community [22], the concept of neurodiversity has since been expanded to include conditions such as attention deficit hyperactivity disorder (ADHD), autism spectrum disorder (ASD), dyslexia, dysgraphia, dyspraxia, dyscalculia and Tourette syndrome, among others. The neurodiversity paradigm offers a balanced approach between the medical and social models for understanding these conditions, defining them through the lens of differences rather than deficits [4,24].

Neurodiverse individuals often experience the world differently due to variations in cognitive and sensory processes, demanding unique considerations in a research setting. Sensory sensitivities, for instance, might be exacerbated by a brightly lit room or loud noises [6]. The necessity to remain still for extended periods of time might also increase anxiety levels, especially in individuals with ADHD or ASD [8,11]. In addition, neurodiverse participants might require more explicit communication due to potential differences in processing information and social cues [5].

Creating an inclusive laboratory environment might contribute not only to the ethical treatment of participants, but also to the accuracy and reliability of research findings [27]. Environmental variables that cause discomfort or heightened anxiety may significantly influence responses in neuroscience experiments [18]. For instance, a review of magnetic resonance imaging (MRI) for autistic patients without sedation or general anesthesia found that adapting communication, optimizing MRI acquisition, and modifying the environment with sensory-easing adjustments were crucial factors for inclusive research [26]. Neglecting to address these factors can lead to the collected data being less reflective of the characteristics of the participants and more indicative of the effect of these variables. As such, taking into consideration the particular needs of neurodiverse participants during the design of experimental protocols may enhance the validity of research studies. However, most existing methodological guidelines consider disabilities at large but do not focus on neurodiversity [17].

To generate these additional guidelines, we engaged with neurodiverse individuals as research advisors who reviewed the entire experimental process, from receiving the invitation email to participating in the test session. We then conducted a 20 min informal interview, inviting them to express their feedback, suggestions, or concerns to contribute to the protocol development. This collaborative process led to the identification of methodological considerations and the amendment of the protocol for our study prior to submission to the institutional ethics review committee (LRS/DP-22/23-35603), ensuring the experimental design was as inclusive as possible.

By sharing our methodological checklist, we hope to assist other researchers in designing laboratory experiments that are inclusive of neurodiverse participants. These guidelines are based on an electroencephalogram (EEG) and eye-tracking study with cognitive tests but could apply to other laboratory tests with similar approaches. Ultimately, each research setting and participant group are unique; therefore, these guidelines are not exhaustive but provide a starting point. Instead of simply copying and pasting this checklist, researchers should consider involving neurodiverse individuals in the planning stages of their projects by forming their own advisory board to review their experimental design and amend their protocol accordingly. An advisory board can help ensure that research practices are as inclusive, ethical, and valid as possible [9].

### Before the experiment

The design of an inclusive experiment starts with recruitment documents. These must be designed with accessibility in mind. For instance, dyslexic individuals often struggle with phonological processing, which can make reading challenging [21]. Those with ADHD may have trouble maintaining focus on large blocks of text, causing their attention to drift before fully comprehending the material [29]. Therefore, the recruitment documents can be made more accessible by breaking up large paragraphs into bullet points or shorter sentences, using simple and clear language, and ensuring high contrast between text and background. To ensure readability and alleviate the potential reading difficulties associated with neurodiversity, our screening survey and information sheet used a beige background with high-contrast fonts [16]. The settings we used were as follows in the online surveying tool [14], but can be adapted for other form builders as well: Background type: Color; Background Color: #F8F3F1; Foreground Contrast: High. Sans serif fonts such as Arial are recommended as they are more accessible [15]. Avoid using all capital letters for continuous text [7].

**Fig. 1:**
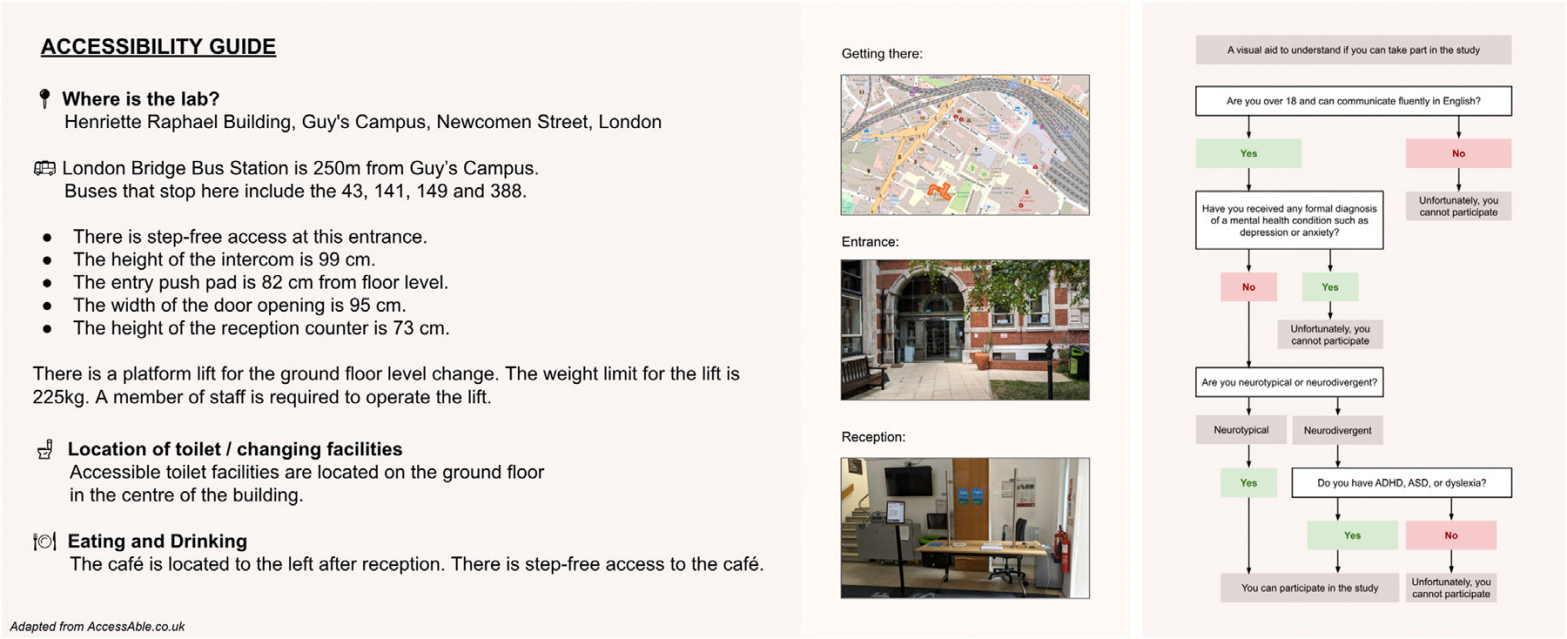
Example accessibility guide for neurodiverse participants.

*Note*: As with recruitment documents, ensure high readability for neurodiverse participants in the invitation documents by using a beige background, high contrast font color, and a sans serif font.

The invitation sent to eligible participants should provide detailed instructions for accessing the laboratory with the exact location of the lab, landmark references, and estimated travel times from popular transport hubs, alongside an accessibility guide to help potential participants find their way to the building, making them aware of the facilities and accommodations available and preparing them for what to expect when they arrive. Such a guide can be created using resources like AccessAble for London-based labs [1], which is the approach our research team took, or they can be built from scratch if no such tool exists where the laboratory is based. At a minimum, the guide should include directions to the lab, photos of the entrance and reception area, information about wheelchair accessibility (steps, lift, ramps, accessible toilets), and details about the availability of eating and drinking facilities, if any (Fig. 1).

When preparing instructions for a neurodiverse group of participants, it is crucial to be as clear, detailed, and multimodal as possible, catering to diverse processing styles. For instance, ASD is associated with a preference for explicit, step-by-step instructions to reduce ambiguity, while those with ADHD may benefit from concise, clearly segmented information to help maintain focus [12,25,28]. Similarly, dyslexic participants might find visual instructions more accessible than written ones due to potential reading difficulties [13]. Some practical suggestions for creating multimodal instructions before the experiment include:

• Designing a visual tree chart to represent the inclusion and exclusion criteria to complement the information sheet (Fig. 1). This entails the creation of a simple flowchart where each branch leads to an outcome (participant is suitable or not suitable). Each node in the flowchart represents a criterion (e.g., age, diagnosis, medication use) so that participants can easily follow the path that applies to them.
• Incorporating a visual guide with nearby landmarks, parking spaces, and public transport stops to complement the written instructions. OpenStreetMap's tagging system can be used to delineate buildings and add notes to guide participants [2].
• Creating an invitation video to complement the email invitation. This could include a virtual tour of the lab, an explanation of what the experiment will involve, and/or a visual demonstration of the tasks that the participant will be asked to complete, depending on the threshold of disclosure necessary to meet the experimental constraints.

Finally, the invitation should include the contact information of the lead researcher or lab assistant to whom they can reach out if they have any questions or concerns, as well as their pronouns, for instance, “she/her”, “he/him”, or “they/them”. It may additionally contain a simple way for the participant to identify the researcher upon entering the premises, thereby reducing the anxiety associated with trying to locate someone they do not know.

### During the experiment

The research environment should foster comfort for all participants as soon as they arrive at the laboratory. While this principle applies to all participants, it takes on added importance for neurodiverse people who may be more susceptible to environmental stressors [6]. Such stressors include overly harsh lighting, loud noises, or uncomfortable temperatures. Other factors could be less tangible, such as a space that feels cramped or the presence of strong odors. For example, one of our research advisors expressed concern about germs, a worry that is common among neurodiverse individuals [20]. Researchers can proactively address this specific concern by clearly indicating that all equipment and surfaces are thoroughly disinfected between test sessions and providing easily accessible hydroalcoholic gel. Other sensory considerations are equally important, with three aspects researchers should keep in mind:

• **Light:** Neurodiverse participants may have an abnormal pupillary light reflex, causing discomfort from bright lights [23]. Dimming the lights prior to starting the experiment can reduce potential distress. Similarly, screen brightness should be adjusted to prevent overstimulation. We found that a screen brightness of 60% and a screen contrast of 65% were most comfortable for participants.
• **Sound:** Sound sensitivity is a common feature in neurodiversity, particularly in ASD where decreased sound tolerance can lead to atypical behavioral responses to environmental sounds [31], and in ADHD where misophonia—the negative emotional response to specific sounds—is often a comorbidity [19]. As such, audio settings should also be carefully calibrated. For instance, we found that a volume level of 70% on a Dell computer was adequate for audibility without causing auditory discomfort for participants with sensory differences. As with brightness and contrast, the audio settings should ideally be adjusted based on feedback from a neurodiverse panel.
• **Temperature:** Research into thermal sensitivity and neurodiversity is scarce, with some studies of in ASD suggesting typical thermal perceptual thresholds [30] and others finding decreased sensitivity to thermal stimuli [3]. Some stimulant medications used to treat ADHD can also affect thermoregulation [10]. If the room has locally controllable air conditioning, researchers should ask the participants about their preferences and adjust the temperature accordingly.

**Fig. 2:**
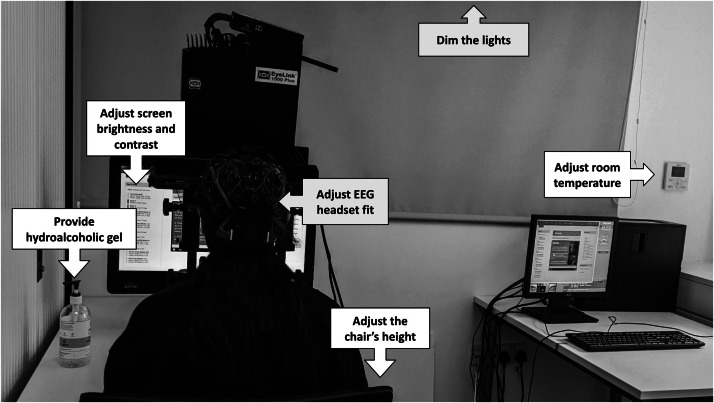
Example of basic adjustments for an experiment involving neurodiverse participants.

Within the constraints of the experimental setup, it can be helpful to actively involve participants in setting up equipment to enhance their comfort and reduce anxiety. For instance, in EEG studies such as ours, researchers can let participants guide the adjustment of the headset to ensure a secure fit without being overly tight—the word our team uses with participants to describe the desired fit is “snug”. Similarly, allowing participants to help adjust the height of the chair when sitting in front of the eye tracker can ensure physical comfort throughout the experiment, which is important for individuals who may have difficulty sitting still for prolonged periods. See Fig. 2 for some of the adjustments to consider during an EEG and eye-tracking experiment.

Proactive communication remains crucial throughout the duration of the experiment. Prior to the start of the experiment, researchers may inquire whether there is any discomfort, potential concerns, or if anything remains unclear. It is also essential to provide a clear roadmap of the experiment, including time estimates for each section, as this can help alleviate participant anxiety and give them a sense of control. For instance, explicitly stating, “This part of the experiment will last for 10 min” or “This is the last part of the experiment” can provide a clear sense of how long each section will take. In between tasks, breaks should be offered to drink water or use the bathroom.

Lastly, fostering an environment that feels calm and orderly can be beneficial. Our research advisors valued an atmosphere that was “quiet and not chaotic,” which highlights the importance of minimizing unnecessary distractions and interruptions. This can be achieved by carefully planning the flow of the experiment to avoid extraneous transitions from one room to another, and maintaining a relaxed, professional demeanor. Similarly, although it is understandable that trainees may need to observe sessions for learning purposes, the presence of multiple observers in the room might feel overwhelming for neurodiverse participants. Limiting the observation to one trainee at a time can be a more sensitive approach. Researchers may also consider scheduling sessions during quieter times, such as early mornings, late afternoons, and weekends, when there is less activity and noise in the laboratory. What is considered distracting can vary from one participant to another—such as a clock ticking, a chair creaking, or a noisy air conditioner—so it can be helpful to directly ask the participant if any distractions remain before starting the experiment.

### After the experiment

The conclusion of the experiment is not merely the end of data collection; rather, it is an opportunity to ensure that participants leave with a positive impression of their involvement, to gather feedback, and to reinforce their connection to the research through follow-up communication. Researchers should pay close attention to the post-experiment phase when working with neurodiverse participants, as this care and consideration can enhance their overall research experience, increase their likelihood of participating in future studies (or of coming back for longitudinal studies), and support dissemination in neurodiverse communities.

Following the experiment's completion, debriefing with participants is critical. This should be more than a routine procedure, as it is the last chance to clarify any questions or concerns the participant may have, ensuring that they depart the lab with a complete understanding of the experiment in which they just participated. Due to the fact that neurodiverse participants may process information differently, researchers should allocate ample time to the debriefing and use simple language that is non-technical and easy to understand.

In addition, it may be beneficial for researchers to solicit feedback from participants regarding their experience. Did they feel comfortable? Was there anything they found challenging? Was there anything that they considered particularly enjoyable? This feedback can be useful in improving future participants' experience in areas not covered by the pre-defined protocol. After their departure, researchers can send an email to participants with information on how to receive the resulting paper when it is published and on how to be alerted to other dissemination outcomes. This keeps participants informed about the outcome of the research to which they contributed. Lastly, the follow-up email should let participants know that they can reach out if they have any further questions or feedback.

## Conclusions

In a laboratory setting, inclusivity extends beyond ordinary ethical considerations. This methodological checklist emphasizes the importance of adopting a neurodiversity-informed approach in research settings and particularly in neuroscience experiments. Central to this perspective is the recognition of neurodiverse individuals not as research subjects but as active contributors to the research process.

Produced in collaboration with a research advisory board of neurodiverse individuals, the proposed checklist focuses on adjusting laboratory-based experiments to be more inclusive and accessible to neurodiverse participants, thus encouraging active and meaningful participation. A multimodal approach to communication, a comfortable environment, and a comprehensive debriefing session can help accommodate the different cognitive and sensory processes of neurodiverse participants, making the experimental process a more positive and less daunting experience. Furthermore, not only is the checklist designed to make neuroscience experiments more accessible and less anxiety-inducing for neurodiverse individuals, but an inclusive research environment reduces the risk of environmental variables skewing data, thus enhancing the validity and reliability of the research.

This checklist is not exhaustive but rather provides a starting point that can be adapted to the specific characteristics of various research settings and participant groups. Involving neurodiverse individuals in the design stage of research can contribute to an inclusive environment. With the adoption of these guidelines and the involvement of neurodiverse individuals in the planning stages, research can progress toward neuroscientific methods that respect and value neurodiversity, leading to a richer understanding of the myriad ways in which the human brain functions.

## Ethics statements

This work did not involve human subjects. Members of the research advisory board who reviewed the experimental design and contributed to creating these methodological guidelines are co-authors of this paper and co-creators of the research.

## CRediT authorship contribution statement

**Anne-Laure Le Cunff:** Conceptualization, Methodology, Writing – original draft. **Caitlin Glover:** Validation, Writing – review & editing. **Brandon-Lee Martis:** Validation, Writing – review & editing. **Vincent Giampietro:** Writing – review & editing, Supervision. **Eleanor Dommett:** Writing – review & editing, Supervision.

## Declaration of competing interest

The authors declare that they have no known competing financial interests or personal relationships that could have appeared to influence the work reported in this paper.

## Data Availability

No data was used for the research described in the article.

## Appendix Supplementary materials

Image, application 1
