# Supplementary material for: Methodological adjustments for experimental studies including neurodiverse participants: A checklist for before, during, and after laboratory visits
Source: MethodsX. 2024 Mar 10;12:102658. doi: 10.1016/j.mex.2024.102658 (PMC10950872; doi:10.1016/j.mex.2024.102658)
Supplement: Supplementary file 1 [file mmc1.pdf]

Checklist for Experimental Studies Including Neurodiverse Participants

Before the experiment

Accessible recruitment documents

- [ ] Break up large paragraphs into bullet points or shorter sentences
- [ ] Use simple and straightforward language
- [ ] Ensure high contrast between text and background (Background type: Color; Background Color: #F8F3F1; Foreground Contrast: High)
- [ ] Use a sans serif font (Arial, Helvetica, Verdana…)
- [ ] Avoid using all capital letters for continuous text

Detailed instructions for accessing the laboratory

- [ ] Directions to the lab
- [ ] Photos of the entrance and reception area
- [ ] Information about wheelchair accessibility (steps, lift, ramps, accessible toilets)
- [ ] Details about the availability of eating and drinking facilities

Multimodal information to cater to diverse processing styles (optional but helpful)

- [ ] Visual tree chart to represent the inclusion and exclusion criteria
- [ ] Visual guide with nearby landmarks, parking spaces, and public transport stops
- [ ] Invitation video to complement the email invitation

During the experiment

Sensory considerations

- [ ] Clearly indicate that all equipment and surfaces are disinfected between test sessions
- [ ] Provide easily accessible hydro-alcoholic gel
- [ ] Adjust the room temperature to a comfortable level
- [ ] Dim the lights to a comfortable level
- [ ] Screen brightness: 60%
- [ ] Screen contrast: 65%
- [ ] Volume level: 70%
- [ ] If the experimental design allows, ensure these levels do not abruptly increase or decrease between task

Proactive communication

- ☐ Where possible, actively involve the participant in setting up the equipment to enhance their comfort and reduce anxiety
- ☐ Before starting the experiment, ask whether there is any discomfort or if anything remains unclear
- ☐ Provide time estimates for each section of the experiment
- ☐ Offer breaks between tasks if the experimental design allows for them

Calm environment

- ☐ Minimize unnecessary distractions
- ☐ Ask the participant if any distractions remain before starting the experiment
- ☐ Avoid extraneous transitions from one room to another
- ☐ Maintain a relaxed, professional demeanor

After the experiment

Debrief

- ☐ Allocate ample time to the debriefing
- ☐ Clarify any questions or concerns the participant may have
- ☐ Use simple language that is non-technical and easy to understand
- ☐ Solicit feedback from participants regarding their experience

Follow-up email

- ☐ Thank the participant for their involvement and feedback
- ☐ Solicit any additional feedback from participants regarding their experience
- ☐ Add a separate email form for the participant to receive the research paper when it is published

Acknowledgments
Created at the Institute of Psychiatry, Psychology & Neuroscience, King's College London, with support from the UKRI Participatory Research Fund.

Authors
Anne-Laure Le Cunff, Caitlin Glover, Brandon-Lee Martis, Vincent Giampietro, Eleanor Dommett.
